# Prospective associations of alcohol and drug misuse with suicidal behaviors among US Army soldiers who have left active service

**DOI:** 10.1017/S0033291725000947

**Published:** 2025-04-28

**Authors:** Laura Campbell-Sills, Xiaoying Sun, Ronald C. Kessler, Robert J. Ursano, Sonia Jain, Murray B. Stein

**Affiliations:** 1 Department of Psychiatry, University of California San Diego, La Jolla, CA, USA; 2 Herbert Wertheim School of Public Health and Human Longevity Science, University of California San Diego, La Jolla, CA, USA; 3 Department of Health Care Policy, Harvard Medical School, Boston, MA, USA; 4 Center for the Study of Traumatic Stress, Department of Psychiatry, Uniformed Services University of the Health Sciences, Bethesda, MD, USA

**Keywords:** alcohol abuse, drug abuse, alcohol use disorder, drug use disorder, substance use disorder, suicidal ideation, suicide attempt, sex differences, military personnel, veterans

## Abstract

**Background:**

This study examines the prospective associations of alcohol and drug misuse with suicidal behaviors among service members who have left active duty. We also evaluate potential moderating effects of other risk factors and whether substance misuse signals increased risk of transitioning from thinking about to attempting suicide.

**Method:**

US Army veterans and deactivated reservists (*N* = 6,811) completed surveys in 2016–2018 (T1) and 2018–2019 (T2). Weights-adjusted logistic regression was used to estimate the associations of binge drinking, smoking/vaping, cannabis use, prescription drug abuse, illicit drug use, alcohol use disorder (AUD), and drug use disorder (DUD) at T1 with suicide ideation, plan, and attempt at T2. Interaction models tested for moderation of these associations by sex, depression, and recency of separation/deactivation. Suicide attempt models were also fit in the subgroup with ideation at T1 (*n* = 1,527).

**Results:**

In models controlling for socio-demographic characteristics and prior suicidality, binge drinking, cannabis use, prescription drug abuse, illicit drug use, and AUD were associated with subsequent suicidal ideation (AORs = 1.42–2.60, *p*s < .01). Binge drinking, AUD, and DUD were associated with subsequent suicide plan (AORs = 1.23–1.95, *p*s < .05). None of the substance use variables had a main effect on suicide attempt; however, interaction models suggested certain types of drug use predicted attempts among those without depression. Additionally, the effects of smoking/vaping and AUD differed by sex. Substance misuse did not predict the transition from ideation to attempt.

**Conclusions:**

Alcohol and drug misuse are associated with subsequent suicidal behaviors in this population. Awareness of differences across sex and depression status may inform suicide risk assessment.

## Introduction

Understanding suicide risk among service members is a priority for military and veterans’ organizations. While significant progress has been made (e.g. Kennedy et al., 2024; Naifeh et al., 2019a; Ruiz et al., 2023), continuing to strengthen the evidence base that guides suicide prevention efforts is critical. Part of this endeavor entails identifying novel risk factors and methods for estimating suicide risk (e.g. Stanley et al., 2022; Stein et al., 2017b). To complement these efforts, investigation is needed to clarify how known risk factors interact and relate to specific outcomes along the continuum of suicidal behavior.

Alcohol and drug misuse have well-documented associations with nonfatal suicidal behaviors and suicide death (Pemau et al., 2024; Poorolajal, Haghtalab, Farhadi, & Darvishi, 2016; Yuodelis-Flores & Ries, 2015), including among those who have served in the military (Ahmed et al., 2023; Bohnert et al., 2017; Campbell-Sills et al., 2019; Fischer et al., 2023; Fuehrlein et al., 2016; Hill et al., 2021; Hoggatt et al., 2015; Kimbrel et al., 2017, 2018; LeardMann et al., 2013; Naifeh et al., 2019b; Polimanti et al., 2021; Stein et al., 2017a). However, questions remain regarding the relationship of substance misuse to suicidal behavior in service members. For instance, recent studies suggest that alcohol and drug misuse may differentiate service members who attempt suicide from those who contemplate but do not act on suicidal thoughts (Langhinrichsen-Rohling, Snarr, Slep, & Heyman, 2019; Nichter et al., 2021). These findings may have important implications for suicide risk assessment and intervention; however, available evidence is based on cross-sectional data. Prospective analyses may help clarify if certain types of substance misuse are risk markers for (as opposed to correlates of) the transition from thinking about to attempting suicide.

Another question pertains to how the associations between substance use behaviors and suicidality outcomes are affected by other risk factors. Females are consistently observed to have increased risks of nonfatal suicidal behaviors, and some evidence suggests that misuse of certain substances (e.g. alcohol and nicotine) is more strongly associated with suicidality in females than in males (Carretta, McKee, & Rhee, 2023; Kittel, Bishop, & Ashrafioun, 2019; Pham et al., 2020; Wilsnack, Wilsnack, Gmel, & Kantor, 2018). Depression is another major risk factor for suicidal behavior that may impact relationships between substance misuse and suicidality outcomes (e.g. Glasheen et al., 2015). Finally, among former service members, a relevant issue to consider is the recency of reintegration into civilian life, as evidence suggests the initial stage of this transition is a high-risk time for suicidal behavior (Chu et al., 2022; Ravindran et al., 2020; Shen, Cunha, & Williams, 2016; Sokol et al., 2021). If other risk factors were found to modify substance use-suicidality associations, this could inform risk assessment and interventions – yet studies of these topics are scarce, and few have focused on service members.

The Army Study to Assess Risk and Resilience in Servicemembers Longitudinal Study (STARRS-LS; Naifeh et al., 2019a) affords an opportunity to investigate the relationships between different forms of substance use and suicidal behaviors among service members. Given concerns about elevated risks of suicidal behavior after military members transition out of active duty, the current study focuses on STARRS-LS respondents who had left the Army (separated or retired) or been released from active duty (deactivated reservists) before participation. We evaluate the prospective associations of different types of substance misuse with suicidal behaviors (ideation, plan, and attempt), including whether they signal an increased risk of transitioning from thinking about to attempting suicide. Additionally, we examine whether other risk factors for suicidal behaviors (sex, depression, and recent reintegration into civilian life) moderate the associations of alcohol and drug misuse with suicidality outcomes. Finally, we include supplementary models to explore how the frequency of substance misuse may relate to the risk of subsequent suicidal behaviors.

## Method

### STARRS-LS overview and participants

STARRS-LS follows a probability sample of service members who completed baseline surveys while on active duty as part of the Army STARRS New Soldier Study, All Army Study, or Pre/Post Deployment Study (Kessler et al., 2013b; Ursano et al., 2014) and agreed to the linkage of their survey responses and Army/Department of Defense (DoD) administrative data. A total of 14,508 current and former US Army soldiers participated in wave 1 (LS1), and 12,156 participated in wave 2 (LS2) of STARRS-LS. The analysis sample for this study was restricted to the 6,811 respondents who were separated, retired, or deactivated for the entire period of interest. This was assessed using survey items that inquired about the respondents’ current military status and when they were last on active duty. Those who reported being on active duty in the 30 days before their LS1 survey or during the interval between their LS1 and LS2 surveys were excluded. The median time since participants were last on active duty was 33 months (IQR = 15–49 months).

STARRS-LS oversampled Army STARRS baseline participants with a history of mental disorders or suicidality, as well as women, National Guard and Reserve members, and Special Operations soldiers. Data collection for LS1 and LS2 occurred from September 2016 to April 2018 and April 2018 to July 2019, respectively. The mean interval between LS1 and LS2 surveys was 17.4 months (SD = 1.4 months); the time between surveys was not related to the study outcomes. Earlier reports provide more details regarding sampling, weighting, and other STARRS-LS procedures (Naifeh et al., 2023; Stanley et al., 2022). Written informed consent was obtained for Army STARRS and STARRS-LS survey participation and for the linkage of survey responses to Army/DoD records. Study procedures were approved by the Institutional Review Boards of the collaborating institutions.

### Measures

#### Suicidality outcomes

The LS2 survey assessed recent suicide ideation, plan, and attempt using items adapted from the Columbia-Suicide Severity Rating Scale (C-SSRS; Posner et al., 2011). The items assessing suicidal ideation asked, ‘Since your last survey in (month/year), how often did you… (a) have thoughts of killing yourself, and (b) wish you were dead or would go to sleep and never wake up?’ Response options were *never, less than once a month, 1–3 days a month, 1–2 days a week, 3–4 days a week*, and *every or nearly every day.* Suicidal ideation was considered present if the respondent indicated *less than once a month* or more to either (a) or (b) or if they reported any suicide plan or attempt since their last survey. That is, even if there was no affirmative response to (a) or (b), the presence of recent suicidal ideation was inferred if the respondent reported a recent suicide plan or suicide attempt as defined below.

The item assessing recent suicide plans asked, ‘Since your last survey in (month/year), how often did you think about how you might kill yourself (e.g. taking pills and shooting yourself) or work out a plan of how to kill yourself?’ followed by the response options listed above. A suicide plan was considered present if the respondent indicated *less than once a month* or more to this item. The item assessing suicide attempts asked, ‘Did you make a suicide attempt (i.e. purposefully hurt yourself with at least some intention to die) at any time since your last survey?’ Response options were *yes* or *no.* For those who answered *yes* or did not respond, a subsequent item asked, ‘How many suicide attempts have you made since your last survey?’ A suicide attempt was considered present if respondents indicated *yes* to the first item or >0 to the second item.

#### Substance use

The LS1 survey evaluated past-30-day substance use with items adapted from the Composite International Diagnostic Interview Screening Scales (CIDI-SC; Kessler & Ustun, 2004). Per the Substance Abuse and Mental Health Services Administration (SAMHSA) definition, Binge Drinking was defined as a response >0 to the item ‘How many days in the past 30 did you have [males: 5 or more; females: 4 or more] drinks containing alcohol within a 24-hour period?’ (0–30 days; item wording was sex-specific). Daily Smoking/Vaping was defined as a response of 30 to the item ‘About how many days in the past 30 did you smoke cigarettes or e-cigarettes?’ (0–30 days). Participants reported frequency of other substance use using the options *never, less than 1 day a week, 1–2 days a week, 3–4 days a week*, and *every or nearly every day.* Cannabis Use was defined as responding *less than 1 day a week* or more to the item asking how often marijuana was used. Prescription Drug Abuse was defined as responding *less than 1 day a week* or more to at least one item assessing abuse of prescription drugs (i.e. ‘a prescription stimulant either without a doctor’s prescription, more than prescribed, or to get high or buzzed’, ‘a prescription tranquilizer or muscle relaxer either without a doctor’s prescription, more than prescribed, or to get high or numbed out’, or ‘a prescription pain reliever to get high, buzzed, or numbed out’). Illicit Drug Use was defined as responding *less than 1 day a week* or more to the item that queried use of ‘any other illegal drug (e.g. cocaine, ecstasy, speed, LSD, and poppers)’.

The LS1 survey also assessed probable alcohol use disorder (AUD) and probable drug use disorder (DUD) with six items adapted from the CIDI-SC that assessed DSM-5 criteria for AUD and DUD (American Psychiatric Association, 2013). The items inquired how often alcohol and drug use caused occupational, interpersonal, and physical/mental health problems; occurred in contexts that could cause harm (e.g., driving); involved excessive amounts of time; or occurred more often or over longer periods of time than intended. Participants reported how often they experienced each problem in the past 30 days using the options *never, 1–3 days a month, 1–2 days a week, 3–4 days a week*, or *every or nearly every day.* AUD and DUD were defined as responding to at least two of the items with a response of *1–2 days a week or more.*

#### Covariates and moderators

Socio-demographic characteristics were used as covariates in all models. They were measured at LS1 and included sex (male or female), age in years, race and ethnicity (Hispanic, Non-Hispanic Black, Non-Hispanic Other, or Non-Hispanic White), marital status [married, never married, or other (separated, divorced, or widowed)], education (high school diploma/GED, some college, or college degree), and military status (separated, retired, or deactivated). Lifetime history of suicide ideation, plan, and attempt at LS1 were also included as covariates as specified in *Data Analysis*; these were assessed with items adapted from the C-SSRS. In terms of possible moderators, we considered sex, past-30-day major depressive episode (MDE) at LS1, and time since active duty (1–12 months or ≥ 13 months). MDE was assessed with items adapted from the CIDI-SC (Kessler et al., 2013a).

### Data analysis

Statistical analyses were conducted in R version 3.6.1 (R Core Team, 2022). Weights were applied in all analyses, which are explained in detail elsewhere (e.g. Naifeh et al., 2023). Briefly, the weights include nonresponse and poststratification adjustments for Army STARRS baseline and STARRS-LS follow-up survey data, as well as adjustments to account for the over-sampling of baseline respondents with certain characteristics (see *STARRS-LS overview and participants*) and the underrepresentation of difficult-to-recruit participants in STARRS-LS.

Weighted prevalence of the substance use, suicidality, and covariate/moderator variables was calculated. Then, a series of weights-adjusted logistic regression models were fit to estimate the associations of each type of substance misuse at LS1 with suicide ideation, plan, and attempt at LS2. All models adjusted for socio-demographic characteristics, military status, and lifetime history of suicidal ideation at LS1. The models of suicide plan at LS2 also adjusted for lifetime suicide plan at LS1, and the models of suicide attempt at LS2 also adjusted for lifetime suicide attempt at LS1. In addition to performing analyses in the full sample (*N* = 6,811), we fit models of suicide attempt at LS2 in the subgroup that reported past-12-month suicidal ideation at LS1 (*n* = 1,527) to evaluate whether the substance misuse variables were associated with the transition from suicide ideation to attempt. We subsequently fit interaction models to test if sex, depression (past-30-day MDE at LS1), or time since active duty moderated the associations of the substance misuse variables with the suicidality outcomes. It was not feasible to evaluate interactions of the hypothesized moderators with Illicit Drug Use and DUD as these were rarely endorsed, and cell sizes for the contrasts of interest were very small. Significant interaction effects were followed up with models stratified by the moderator in question. Two-tailed *p* values <.05 were considered statistically significant.

#### Supplementary models

To investigate possible ‘dose–response’ effects of substance misuse on suicide-related outcomes, we fit models that replaced the binary variables reflecting Binge Drinking, Smoking/Vaping, Cannabis Use, Prescription Drug Abuse, and Illicit Drug Use at LS1 with ordinal variables that captured frequency of these types of substance misuse at LS1 (e.g. frequent *vs* infrequent *vs* no episodes). The results of these models are described briefly in the ‘Results’ Section, with more details provided in the Supplementary Material.

## Results

Table 1 displays the distribution of socio-demographic, substance misuse, and clinical characteristics at LS1. Sample characteristics stratified by military status (separated, retired, or deactivated) are shown in Supplementary Table 1. The veterans and deactivated reservists were predominantly male (82.2%) with a mean age of 30.7 (SD = 8.3) years at LS1. Most (55.1%) were separated from service, with another 26.4% deactivated and 18.5% retired. In terms of the history of suicidal behaviors at LS1, 38.1%, 22.7%, and 7.3% reported lifetime ideation, plan, and attempt, respectively. Binge Drinking was the most common form of substance misuse, with a prevalence of 32.8%. Daily Smoking/Vaping (17.3%), Cannabis Use (10.4%), and AUD (8.7%) were also common. To illustrate the persistence of different forms of substance misuse during the study period, Supplementary Table 2 shows the prevalence of substance misuse at LS2 stratified by substance misuse at LS1.

**Table 1.** Sample characteristics at wave 1 of STARRS-LS (overall and stratified by sex)

	Overall (*N* = 6,811)			Male (*n* = 5,655)			Female (*n* = 1,226)		
	*N*	%	SE	*N*	%	SE	*N*	%	SE
Sex									
Male	5,655	82.2	0.8	N/A	N/A	N/A	N/A	N/A	N/A
Female	1,226	17.8	0.8	N/A	N/A	N/A	N/A	N/A	N/A
Age (mean, SD)	6,811	30.7	8.3	5,655	30.8	8.3	1,226	29.6	7.8
Race and ethnicity									
Non-Hispanic White	4,820	67.3	1.0	4,144	71.0	1.1	676	50.2	2.2
Non-Hispanic Black	867	15.4	0.7	561	12.6	0.7	306	28.7	2.1
Hispanic	776	11.2	0.6	605	10.5	0.6	171	14.6	1.5
Non-Hispanic Other	418	6.1	0.6	345	6.0	0.7	73	6.5	0.7
Education									
HS diploma/GED	2,791	41.5	1.0	2,293	41.6	1.0	498	41.0	2.1
Some college	2,225	33.2	0.8	1,867	33.8	0.9	358	30.4	2.0
College degree	1,822	25.3	0.9	1,461	24.6	1.0	361	28.6	1.8
Marital status									
Married	3,346	47.8	1.1	2,823	49.3	1.2	523	40.9	1.8
Never married	2,492	37.0	1.0	2,020	36.4	1.1	472	39.8	2.1
Divorced/separated/widowed	1,035	15.2	0.7	807	14.3	0.8	228	19.3	1.4
Military status									
Deactivated Guard/Reserve	2,201	26.4	0.9	1,780	26.3	0.9	421	26.8	1.6
Separated	3,557	55.1	1.0	2,902	54.4	1.1	655	58.2	2.5
Retired	1,123	18.5	0.9	973	19.3	0.9	150	15.0	2.1
Suicidal ideation (lifetime)	3,050	38.1	1.1	2,502	37.0	1.3	548	43.3	1.9
Suicide plan (lifetime)	1,671	22.7	0.8	1,361	22.0	0.9	310	26.1	1.6
Suicide attempt (lifetime)	542	7.3	0.5	410	6.4	0.5	132	11.6	1.4
Major depression (past 30 days)	1,447	21.9	1.2	1,150	20.7	1.3	297	27.6	1.9
Binge drinking (past 30 days)	2,352	32.8	0.9	2,040	34.4	1.1	312	25.6	1.8
Daily smoking/vaping (past 30 days)	1,135	17.3	0.9	992	18.2	1.1	143	12.9	1.5
Cannabis use (past 30 days)	751	10.4	0.7	640	10.4	0.7	111	10.7	1.4
Illicit drug use (past 30 days)	110	1.5	0.3	94	1.3	0.2	16	2.5	1.1
Prescription drug abuse (past 30 days)	239	3.4	0.4	191	3.3	0.4	48	4.2	1.2
Alcohol use disorder (past 30 days)	637	8.7	0.5	552	9.0	0.6	85	7.1	1.0
Drug use disorder (past 30 days)	151	1.9	0.2	129	1.9	0.3	22	2.0	0.6

*Note*: All means and percentages are weighted.

### Prospective associations of alcohol and drug misuse with suicidality outcomes

Table 2 shows the results of models evaluating the associations of past-30-day substance misuse at LS1 with suicidality outcomes at LS2, adjusting for socio-demographic characteristics, military status, and prior history of suicidality. At LS2, 27.8% of the veterans and deactivated reservists reported suicidal ideation since their last survey. Binge Drinking (AOR = 1.42, 95% CI = 1.19–1.70, *p* < .0005), Cannabis Use (AOR = 1.46, 95% CI = 1.11–1.91, *p* = .007), Prescription Drug Abuse (AOR = 1.99, 95% CI = 1.23–3.23, *p* = .005), Illicit Drug Use (AOR = 2.60, 95% CI = 1.38–4.91, *p* = .003), and AUD (AOR = 2.24, 95% CI = 1.73–2.90, p < .0005) at LS1 were associated with increased odds of suicidal ideation at LS2. Daily Smoking/Vaping (AOR = 1.11, 95% CI = 0.82–1.51, *p* = .50) and DUD (AOR = 1.72, 95% CI = 0.96–3.08, *p* = .069) were not significantly associated with suicidal ideation at LS2. The supplementary models that examined substance use frequency in relation to the risk of ideation provided some evidence of dose–response effects (see Supplementary Table 3). The associations of Binge Drinking and Cannabis Use with suicidal ideation were more pronounced in those reporting more frequent episodes and, in the case of Cannabis Use, only statistically significant in those reporting at least weekly use. In contrast, the associations of Prescription Drug Abuse and Illicit Drug Use with the risk of ideation were not more pronounced in those reporting more versus less frequent use.

**Table 2.** Prospective associations of alcohol and drug misuse with suicidal behaviors among US Army veterans and deactivated reservists

Substance misuse at wave 1 of STARRS-LS	Outcomes at wave 2 of STARRS-LS			
	Suicidal ideation	Suicide plan	Suicide attempt (full sample)	Suicide attempt (among ideators)
Binge drinking	1.42 (1.19–1.70)***	1.23 (1.01–1.51)*	1.07 (0.64–1.78)	1.10 (0.59–2.04)
Daily smoking/vaping	1.11 (0.82–1.51)	0.86 (0.65–1.13)	1.35 (0.75–2.43)	1.23 (0.62–2.44)
Cannabis use	1.46 (1.11–1.91)**	1.04 (0.72–1.52)	1.25 (0.63–2.48)	0.68 (0.31–1.50)
Prescription drug abuse	1.99 (1.23–3.23)**	1.50 (0.92–2.46)	1.64 (0.71–3.81)	0.84 (0.30–2.36)
Illicit drug use	2.60 (1.38–4.91)**	1.12 (0.52–2.39)	1.83 (0.50–6.68)	1.53 (0.37–6.29)
Alcohol use disorder	2.24 (1.73–2.90)***	1.41 (1.08–1.85)*	1.33 (0.68–2.62)	1.10 (0.53–2.26)
Drug use disorder	1.72 (0.96–3.08)	1.95 (1.09–3.49)*	1.15 (0.40–3.31)	0.59 (0.17–2.10)

*Note*: Full sample models are based on data from the 6,811 respondents who met study inclusion criteria. Subgroup models (‘among ideators’) are based on data from 1,527 respondents who reported past-12-month suicidal ideation at LS1. The models estimate the main effect of each substance misuse variable (one at a time) on each suicidality outcome, controlling for sex, age, race and ethnicity, marital status, education, military status (separated, deactivated, or retired), and lifetime history of suicidal ideation at LS1. Suicide plan models also control for a lifetime history of suicide plan at LS1, and suicide attempt models also control for a lifetime history of suicide attempt at LS1. Some of the associations reported here are qualified by interactions reported in the ‘Results’ Section and figures. *p < .05, **p < .01, ***p < .001.

At LS2, 15.5% of the veterans and deactivated reservists reported contemplating a suicide plan since their last survey. Only Binge Drinking (AOR = 1.23, 95% CI = 1.01–1.51, *p* = .045), AUD (AOR = 1.41, 95% CI = 1.08–1.85, *p* = .012), and DUD (AOR = 1.95, 95% CI = 1.09–3.49, *p* = .025) at LS1 were associated with increased odds of suicide plan at LS2. Results of the supplementary analysis suggested that the risk of subsequent suicide plan was only significantly increased among those reporting regular alcohol binges (≥ 5 in the past 30 days; see Supplementary Table 3).

At LS2, 1.4% of the full sample and 4.9% of those with past-12-month suicidal ideation at LS1 reported that they had made a suicide attempt since their last survey. As shown in Table 2, none of the forms of substance misuse at LS1 was associated with suicide attempt at LS2 in the full sample (all *p* > .24) or in the subsample with past-12-month ideation at LS1 (all *p* > .34).

### Moderation of substance misuse-suicidality associations by other risk factors

**Figure 1.**
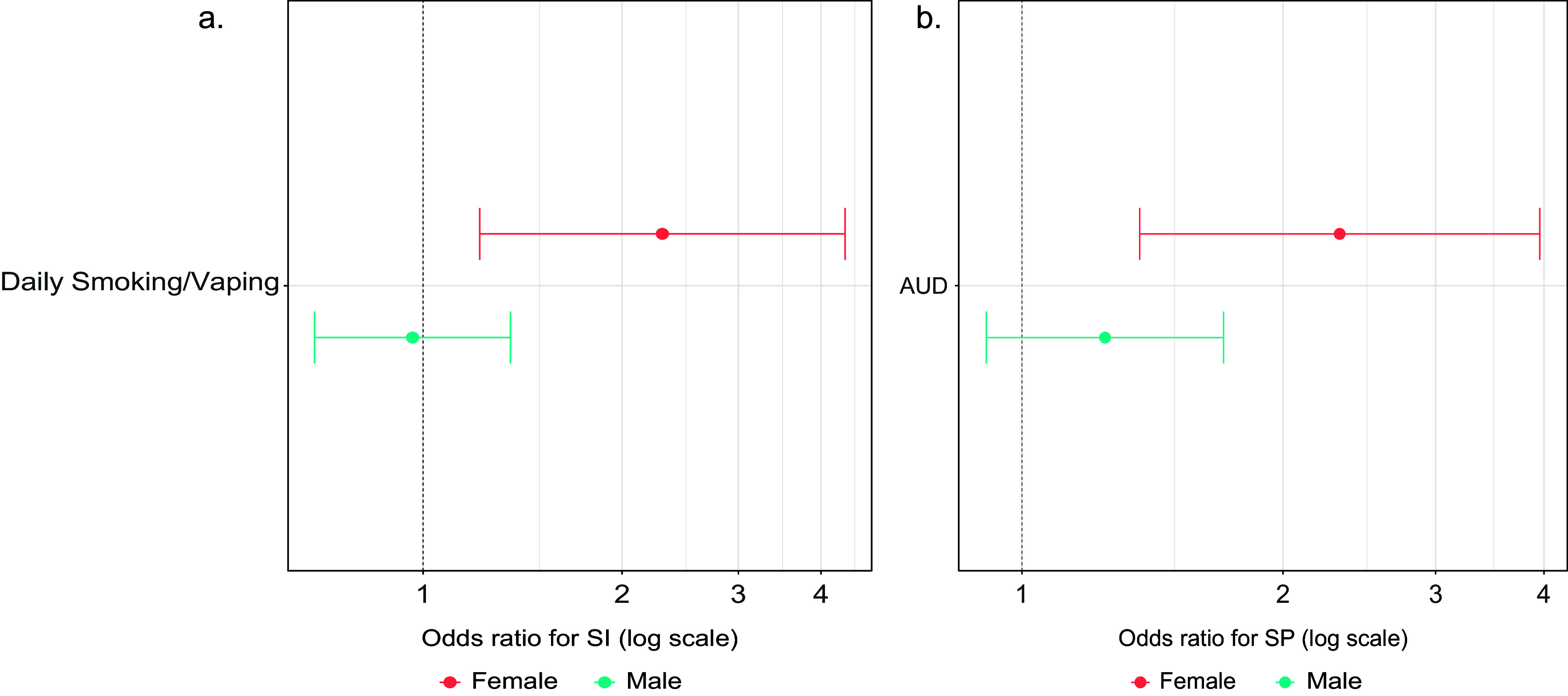
Results of sex-stratified models of the associations of (a) Daily Smoking/Vaping at LS1 with suicidal ideation at LS2 and (b) AUD at LS1 with suicide plan at LS2. The models adjusted for age, race and ethnicity, marital status, education, military status, lifetime history of suicidal ideation at LS1, and (for the model depicted in panel b) lifetime history of suicide plan at LS1. AUD, alcohol use disorder; SI, suicidal ideation; SP, suicide plan.

Supplementary Table 4 shows the analyses of sex differences in the associations between substance misuse at LS1 and suicidal behaviors at LS2. Sex was a significant moderator of the relationship between Daily Smoking/Vaping and subsequent suicidal ideation (AOR = 2.29, 95% CI = 1.06–4.94, *p* = .034 for Sex × Daily Smoking/Vaping), with stratified models indicating that Daily Smoking/Vaping was associated with increased odds of ideation in females (AOR = 2.30, 95% CI: 1.22–4.35, *p* = .010), but not in males (AOR = 0.96, 95% CI: 0.69–1.36, *p* = .83; see panel a of Figure 1). Sex also moderated the association between AUD and subsequent suicide plan (AOR = 1.91, 95% CI = 1.04–3.50, *p* = .037 for Sex × AUD), with stratified models showing that AUD was associated with increased odds of plan in females (AOR = 2.33, 95% CI = 1.37–3.96, *p* = .002), but not in males (AOR = 1.25, 95% CI = 0.91–1.71, *p* = .17; see panel b of Figure 1).

**Figure 2.**
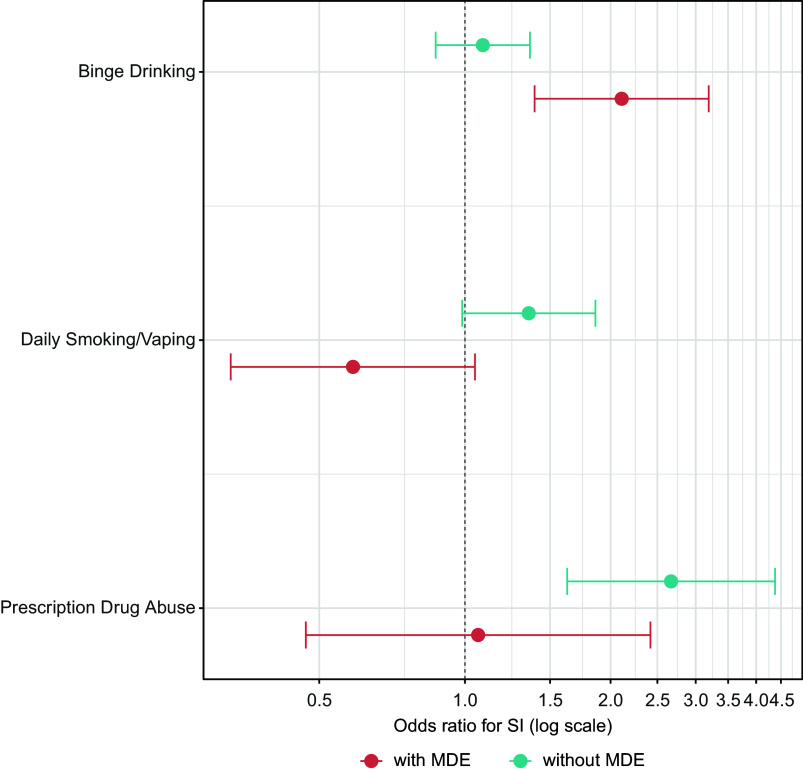
Results of MDE-stratified models of the associations of Binge Drinking, Daily Smoking/Vaping, and Prescription Drug Abuse at LS1 with suicidal ideation at LS2. The models adjusted for sex, age, race and ethnicity, marital status, education, military status, and lifetime history of suicidal ideation at LS1. MDE, major depressive episode; SI, suicidal ideation.

Supplementary Table 5 shows the results of models evaluating the moderating effects of co-occurring MDE. Multiple interactions were observed, with only one reflecting synergistic effects of MDE and substance misuse. This involved an MDE x Binge Drinking effect on the odds of suicidal ideation (AOR = 1.78, 95% CI = 1.09–2.91, *p* = .021). Stratified models showed Binge Drinking was associated with later suicidal ideation in respondents with MDE (AOR = 2.11, 95% CI: 1.39–3.19, *p* < .0005) but not in those without MDE (AOR = 1.09, 95% CI: 0.87–1.36, *p* = .46); see Figure 2. Given the synergistic nature of the interaction, the combination of MDE and Binge Drinking was associated with more than four times the odds of later ideation (2.32 * 1.11 * 1.78 = 4.58; see Supplementary Table 5), relative to having neither risk factor.

**Figure 3.**
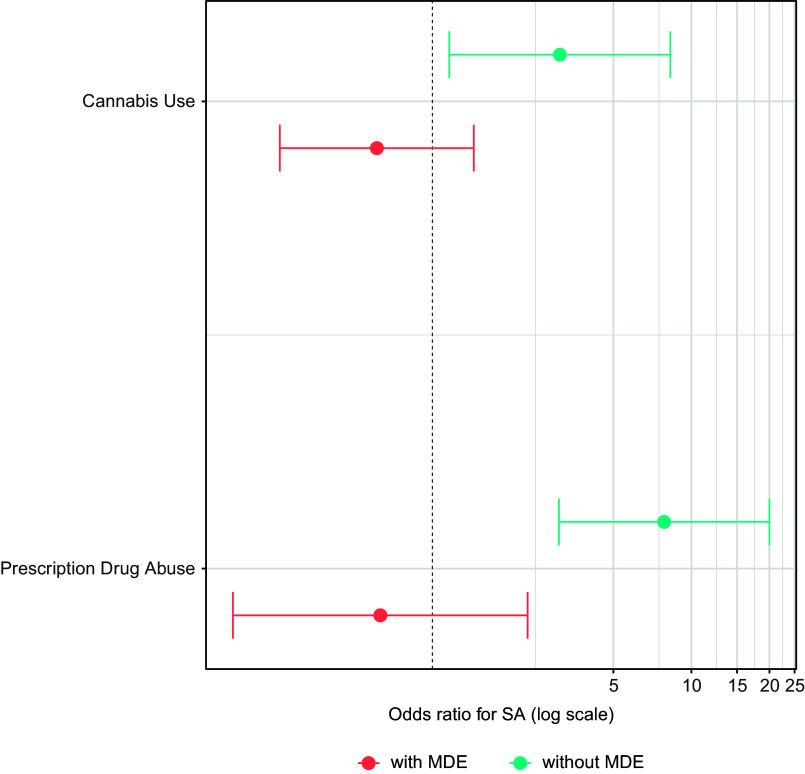
Results of MDE-stratified models of the associations of Cannabis Use and Prescription Drug Abuse at LS1 with suicide attempt at LS2. The models adjusted for sex, age, race and ethnicity, marital status, education, military status, lifetime history of suicidal ideation at LS1, and lifetime history of suicide attempt at LS1. MDE, major depressive episode; SA, suicide attempt.

Also notable was the finding that MDE moderated the associations of Prescription Drug Abuse with all three suicidality outcomes (ideation: AOR = 0.38, 95% CI = 0.15–0.96, *p* = .041; plan: AOR = 0.31, 95% CI = 0.13–0.75, *p* = .009; attempt: AOR = 0.10, 95% CI = 0.02–0.49, *p* = .005). Prescription Drug Abuse was associated with increased odds of each outcome in those without MDE (ideation: AOR = 2.67, 95% CI: 1.63–4.38, *p* < .0005; plan: AOR = 2.62, 95% CI: 1.53–4.51, *p* < .0005; attempt: AOR = 7.85, 95% CI: 3.08–20.00, *p* < .0005) but not in those with MDE (ideation: AOR = 1.07, 95% CI: 0.47–2.42, *p* = .88; plan: AOR = 0.83, 95% CI: 0.41–1.68, *p* = .60; attempt: AOR = 0.63, 95% CI: 0.17–2.33, *p* = .49). See Figures 2 and 3. Evidence from the interaction models also indicated increased risks of suicide attempts associated with Cannabis Use, specifically among those without MDE (Figure 3), as well as differences in the associations of Daily Smoking/Vaping with suicide ideation (Figure 2) and plan (Supplementary Table 5) based on MDE status.

The final potential moderator we considered was time since active duty (1–12 months vs ≥13 months; see Supplementary Table 6). Time since active duty did not significantly modify the associations of Binge Drinking, Daily Smoking/Vaping, Cannabis Use, Prescription Drug Use, or AUD at LS1 with the suicidality outcomes at LS2 in the full sample. Additionally, none of the hypothesized moderators exhibited significant interaction effects with the substance use variables in the subgroup models of suicide attempt at LS2 among those with past-12-month ideation at LS1 (see Supplementary Table 7).

## Discussion

In a large sample of Army veterans and deactivated reservists, most forms of alcohol and drug misuse were associated with later suicidal ideation when controlling for the effects of socio-demographic characteristics and previous history of suicidal ideation. Binge Drinking, AUD, and DUD were also associated with increased risk of subsequent suicide plan, while excess risks of suicide attempt related to substance misuse were limited to those without concurrent depression. Contrary to expectations, our results did not indicate that substance misuse was a risk marker for the transition from thinking about suicide to making an attempt.

Several findings regarding alcohol misuse and suicidality risk are noteworthy. Binge drinking was the most common form of substance misuse, reported by approximately one-third of veterans and deactivated reservists; AUD also had a relatively high prevalence at nearly 9%. Both were associated with subsequent suicide ideation and plan, although a more fine-grained analysis revealed differences in these associations based on other factors. With respect to binge drinking, the excess risk of suicidal ideation was confined to respondents with concurrent MDE, among whom binge drinking was associated with approximately twice the odds of later ideation. Additionally, analyses that probed dose–response effects suggested that a significantly elevated risk of subsequent suicide plan was only apparent in veterans and deactivated reservists who reported regular alcohol binges (at least five in the past 30 days, or ‘heavy alcohol use’ according to SAMHSA). Beyond other potential harms associated with binge drinking in this population (Stahre, Brewer, Fonseca, & Naimi, 2009), the current results suggest that binge drinking during a depressive episode indicates a substantially elevated risk of suicidal ideation. Moreover, regular binge drinking (i.e. weekly or more) may signal an increased risk of more serious thoughts of suicide that involve contemplating a plan.

In contrast to the findings for binge drinking, AUD was associated with an increased risk of later suicidal ideation irrespective of depression status. Given the robust effects of AUD and MDE on the risk of suicidal ideation – and their apparent additivity – maintaining vigilance for the emergence of suicidal thoughts among those with co-occurring AUD and MDE is crucial. Among female veterans and deactivated reservists, AUD may also signal an elevated risk of developing a suicide plan. The models indicated that, while AUD did not predict suicide plan in males, it was associated with more than twice the odds of subsequent suicide plan in females. This finding – along with our observation that daily smoking/vaping was associated with more than twice the odds of subsequent suicidal ideation in females (but not males) – converges with other evidence that alcohol and nicotine misuse may lead to worse outcomes in females (Kittel et al., 2019; Pham et al., 2020). Collectively, these findings also reinforce the importance of continued investigation of potential sex differences in sequelae of alcohol and drug misuse.

Cannabis use was also relatively common, with a prevalence of 10%. Cannabis use was associated with subsequent suicidal ideation, with the dose–response analysis suggesting that the increased risk of ideation was confined to those who reported using cannabis at least weekly. In contrast, illicit drug use was rarely reported (prevalence of 1.5%) and associated with subsequent suicidal ideation even when use was infrequent (less than weekly). Given the low endorsement of illicit drug use in this study, we may have been limited in our ability to detect dose–response effects. Future studies should reevaluate this issue as well as the possibility of moderation of the associations of illicit drug use and suicidal behaviors by other risk factors (which was deemed infeasible given the low endorsement of illicit drug use in this study).

A final contribution of the current study is the identification of potential risk factors for suicidal thoughts and behavior among individuals without depression. Substantial proportions of service members who attempt or die from suicide have never been diagnosed with depression or other mental disorders (Britton et al., 2012; Ursano et al., 2018). We found that prescription drug abuse was a significant predictor of all suicidality outcomes in service members without MDE. Among respondents without past-30-day MDE, those who reported prescription drug abuse were estimated to have more than 2.5 times the odds of subsequent suicide ideation and planning, and nearly eight times the odds of later suicide attempt, relative to those who denied prescription drug abuse. Cannabis use was also associated with increased odds of suicide attempts among those without MDE, although this effect was less pronounced than that of prescription drug abuse.

### Limitations

This study has several noteworthy limitations. First, while the sample size is relatively large, the analysis may still be underpowered to detect prospective effects of substance misuse on suicide attempts because that outcome is rare. This issue may have been compounded in models where the predictor was also infrequent, resulting in small cell sizes for the contrasts of interest (e.g. illicit drug use and DUD were rarely reported). Another limitation is that our study relied on self-reports of substance use, depressive symptoms, and suicidal behaviors. These data are vulnerable to response bias, including the likelihood that some respondents underreported their alcohol and drug use. Third, the assessment of cannabis use did not distinguish between medicinal and recreational use, and these contexts have different effects on the risk of suicidal behaviors. Fourth, some potentially important dimensions of substance use were not captured in the independent variables used in our analysis (e.g. persistence/worsening and typical quantity consumed), and these factors should be examined in future work. Finally, while our study examined potential moderation by recency of reintegration, we did not examine other service characteristics (e.g. deployment history and discharge characterization) that might affect the associations between substance misuse and subsequent suicidal behaviors.

In summary, alcohol and drug misuse were associated with subsequent suicidal behaviors among US Army veterans and deactivated reservists. Some of the substance misuse-suicidality associations depended on other factors, such as the participant’s sex or depression status. Additionally, excess risks of suicidal behaviors associated with binge drinking and cannabis use were typically confined to those who reported frequent episodes (weekly or more). Awareness of interactions between substance misuse and other risk factors may inform efforts to stratify suicide risk in this population. These findings also reinforce the importance of continued investigation of the complex interplay between substance misuse and other risk factors for suicidal behavior.

## Supporting information

Campbell-Sills et al. supplementary material Campbell-Sills et al. supplementary material

## Supplementary material

The supplementary material for this article can be found at http://doi.org/10.1017/S0033291725000947.
