# Supplementary material for: Prospective associations of alcohol and drug misuse with suicidal behaviors among US Army soldiers who have left active service
Source: Psychol Med. 2025 Apr 28;55:e119. doi: 10.1017/S0033291725000947 (PMC12094637; doi:10.1017/S0033291725000947)
Supplement: Campbell-Sills et al. supplementary material [file S0033291725000947sup001.pdf]

Supplementary Table 1: Sample characteristics at wave 1 of STARRS-LS stratified by military status

	Deactivated (n=2201)			Separated (n=3557)			Retired (n=1123)		
	N	%	SE	N	%	SE	N	%	SE
Sex									
Male	1780	82.1	1.1	2902	81.3	1.1	973	85.7	1.8
Female	421	17.9	1.1	655	18.7	1.1	150	14.3	1.8
Age (mean, SD)	2201	28.7	6.9	3557	28.4	5.4	1123	40.5	9.8
Race and ethnicity									
Non-Hispanic White	1494	65.2	1.5	2533	69	1.5	793	65.2	2.5
Non-Hispanic Black	286	15.8	1.2	431	14.1	0.9	150	18.8	2.3
Hispanic	269	12.6	1	390	10.6	0.8	117	11	1.3
Non-Hispanic Other	152	6.4	0.7	203	6.2	0.9	63	5.1	1.2
Education									
HS diploma/GED	878	42.8	1.5	1611	46.7	1.2	302	24.4	2.3
Some college	669	31.7	1.4	1186	34.6	1.2	370	31.1	2.3
College degree	643	25.5	1.4	733	18.7	1	446	44.5	2.5
Marital status									
Married	978	43.6	1.6	1557	42	1.4	811	71.1	2.7
Never married	1013	47.6	1.6	1363	40.8	1.4	116	10.7	1.9
Divorced/separated/widowed	207	8.8	0.8	633	17.2	1.1	195	18.2	2.1
Lifetime suicidal ideation	759	28.6	1.3	1727	41.2	1.5	564	42.5	2.6
Lifetime suicide plan	353	14.4	1	974	25.6	1.2	344	25.8	2
Lifetime suicide attempt	105	4	0.5	318	8.6	0.6	119	8.3	1.4
Major depression (past 30 days)	203	9.3	0.8	848	23.7	1.4	396	34.6	3
Binge drinking (past 30 days)	677	30.1	1.4	1353	36.7	1.2	322	25.4	2.1
Daily smoking/vaping (past 30 days)	248	11.8	1.1	706	20.5	1.3	181	15.7	2.3
Cannabis use (past 30 days)	54	2.3	0.5	571	15.2	1	126	7.9	1.2
Illicit drug use (past 30 days)	11	0.5	0.2	86	2	0.3	13	1.7	1
Prescription drug abuse (past 30 days)	33	1.5	0.3	156	4.1	0.5	50	4.3	1.2
Alcohol use disorder (past 30 days)	126	5	0.7	414	10.9	0.8	97	7	1.1
Drug use disorder (past 30 days)	16	0.6	0.2	111	2.6	0.4	24	1.8	0.6

Note. Means and %s reported in the table are weighted. Military status was self-reported at wave 1 of STARRS-LS.

Supplementary Table 2: Cross-tabulation of substance misuse at wave 1 (LS1) and wave 2 (LS2) of STARRS-LS

	Binge Drinking at LS2 - NO		Binge Drinking at LS2- YES		Total	
Binge Drinking at LS1 - NO	3728	83.51%	736	16.49%	4464	100%
Binge Drinking at LS1 - YES	837	35.78%	1502	64.22%	2339	100%
Total	4565	67.10%	2238	32.90%	6803	100%

	Smoking/Vaping at LS2 - NO		Smoking/Vaping at LS2 - YES		Total	
Smoking/Vaping at LS1 - NO	5416	95.44%	259	4.56%	5675	100%
Smoking/Vaping at LS1 - YES	285	25.27%	843	74.73%	1128	100%
Total	5701	83.80%	1102	16.20%	6803	100%

	Cannabis Use at LS2 - NO		Cannabis Use at LS2 - YES		Total	
Cannabis Use at LS1 - NO	5656	93.03%	424	6.97%	6080	100%
Cannabis Use at LS1 - YES	263	35.16%	485	64.84%	748	100%
Total	5919	86.69%	909	13.31%	6828	100%

	Illicit Drug Use at LS2 - NO		Illicit Drug Use at LS2 - YES		Total	
Illicit Drug Use at LS1 - NO	6572	98.41%	106	1.59%	6678	100%
Illicit Drug Use at LS1 - YES	73	66.36%	37	33.64%	110	100%
Total	6645	97.89%	143	2.11%	6788	100%

	Rx Drug Abuse at LS2 - NO		Rx Drug Abuse at LS2 - YES		Total	
Rx Drug Abuse at LS1 - NO	6476	97.66%	155	2.34%	6631	100%
Rx Drug Abuse at LS1 - YES	166	69.46%	73	30.54%	239	100%
Total	6642	96.68%	228	3.32%	6870	100%

	Alcohol Use Disorder at LS2 - NO		Alcohol Use Disorder at LS2 - YES		Total	
Alcohol Use Disorder at LS1 - NO	5812	93.08%	432	6.92%	6244	100%
Alcohol Use Disorder at LS1 - YES	308	48.35%	329	51.65%	637	100%
Total	6120	88.94%	761	11.06%	6881	100%

	Drug Use Disorder at LS2 - NO		Drug Use Disorder at LS2 - YES		Total	
Drug Use Disorder at LS1 - NO	6559	97.46%	171	2.54%	6730	100%
Drug Use Disorder at LS1 - YES	87	57.62%	64	42.38%	151	100%
Total	6646	96.58%	235	3.42%	6881	100%

Note. The %s reported are unweighted row %s for the contingency tables.

Supplementary Table 3: Supplementary analysis examining dose-response effects in substance misuse-suicidality assocations

			Suicidal ideation at LS2			Suicide plan at LS2			Suicide attempt at LS2		
	Level of Use	n	AOR	CI lower	CI upper	AOR	CI lower	CI upper	AOR	CI lower	CI upper
Past-30-day binge drinking at LS1	0 binges	4503	ref	ref	ref	ref	ref	ref	ref	ref	ref
	1-4 binges	1537	**1.29**	**1.04**	**1.60**	1.07	0.82	1.40	0.87	0.50	1.52
	5 or more binges	815	**1.65**	**1.25**	**2.18**	**1.49**	**1.13**	**1.97**	1.32	0.65	2.67
Past 30-day smoking/vaping at LS1	0 days	5045	ref	ref	ref	ref	ref	ref	ref	ref	ref
	1-29 days	678	1.10	0.83	1.46	1.15	0.80	1.64	0.89	0.36	2.17
	30 days	1135	1.13	0.84	1.53	0.88	0.66	1.17	1.32	0.72	2.44
Past-30-day cannabis use at LS1	Never	6101	ref	ref	ref	ref	ref	ref	ref	ref	ref
	<1x/week	239	1.25	0.84	1.86	1.06	0.69	1.64	1.43	0.52	3.93
	1x/week or more	512	**1.56**	**1.10**	**2.21**	1.03	0.64	1.68	1.17	0.49	2.80
Past-30-day Illicit Drug Use at LS1	Never	6723	ref	ref	ref	ref	ref	ref	ref	ref	ref
	<1x/week	79	**3.22**	**1.64**	**6.31**	0.69	0.24	1.94	0.85	0.11	6.38
	1x/week or more	31	1.61	0.45	5.83	**3.49**	**1.34**	**9.07**	4.28	0.86	21.34
Past-30-day Prescription Drug Abuse at LS1	Never	6636	ref	ref	ref	ref	ref	ref	ref	ref	ref
	<1x/week	108	**1.92**	**1.15**	**3.19**	**1.84**	**1.05**	**3.24**	1.66	0.54	5.10
	1x/week or more	131	**2.03**	**1.02**	**4.02**	1.36	0.71	2.61	1.64	0.55	4.86

Note: The table shows adjusted odds ratios (AORs) with 95% confidence intervals (CIs). The models adjust for socio-demographic characteristics, military status, and lifetime suicidality as described in Data Analysis. Significant adjusted odds ratios are shown in bold. For substances assessed on a 5-point response scale, responses reflecting no use (*Never*) were contrasted with those indicating infrequent use *(less than 1x/week)* and frequent use (*1-2 days/week* to *every or nearly every day*). Binge Drinking was assessed as number of days with alcohol binges. We chose to contrast 0 days with 1-4 days and 5 or more days, given that the Substance Abuse and Mental Health Services Administration defines Heavy Alcohol Use as 5 or more alcohol binges in a 30 day period. Finally, as daily use was the most frequent pattern among nicotine users, we contrasted no use (0 days) with 1-29 days and with 30 days.

Supplementary Table 4: Models of wave 1 substance use x sex interaction effects on suicidal behaviors at wave 2

	Suicidal ideation			Suicide Plan			Suicide attempt		
	AOR	CI lower	CI upper	AOR	CI lower	CI upper	AOR	CI lower	CI upper
Binge Drinking	1.43	1.17	1.75	1.18	0.94	1.47	1.00	0.54	1.85
Female	1.20	0.90	1.61	1.00	0.70	1.42	1.58	0.75	3.36
Binge Drinking x Female	0.96	0.60	1.56	1.28	0.75	2.18	1.23	0.36	4.17
Daily Smoking/Vaping	0.98	0.70	1.38	0.86	0.63	1.16	1.59	0.81	3.10
Female	1.00	0.79	1.28	1.04	0.77	1.42	1.98	1.01	3.89
Daily Smoking/Vaping x Female	**2.29**	**1.06**	**4.94**	1.03	0.48	2.23	0.54	0.13	2.14
Cannabis Use	1.45	1.07	1.97	0.99	0.63	1.55	1.16	0.50	2.67
Female	1.15	0.89	1.48	1.02	0.73	1.41	1.59	0.79	3.18
Cannabis Use x Female	1.02	0.50	2.09	1.28	0.55	2.96	1.25	0.28	5.57
Prescription Drug Abuse	1.71	1.00	2.94	1.45	0.87	2.42	1.40	0.55	3.56
Female	1.11	0.86	1.42	1.04	0.77	1.41	1.58	0.83	3.03
Prescription Drug Abuse x Female	2.23	0.82	6.08	1.17	0.28	4.84	1.49	0.24	9.27
Alcohol Use Disorder	2.05	1.53	2.73	1.26	0.93	1.72	1.29	0.58	2.90
Female	1.12	0.87	1.44	0.98	0.72	1.35	1.66	0.83	3.31
Alcohol Use Disorder x Female	1.84	0.98	3.45	**1.91**	**1.04**	**3.50**	1.11	0.22	5.49

Note: The table shows adjusted odds ratios (AORs) with 95% confidence intervals (CIs). Models adjust for socio-demographic characteristics, military status, and lifetime suicidality as described in Data Analysis. Significant interaction effects are shown in bold.

Supplementary Table 5: Models of wave 1 substance use x MDE interaction effects on suicidal behaviors at wave 2

	Suicidal ideation			Suicide Plan			Suicide attempt		
	AOR	CI lower	CI upper	AOR	CI lower	CI upper	AOR	CI lower	CI upper
Binge Drinking	1.11	0.89	1.38	1.23	0.89	1.69	0.86	0.41	1.78
MDE	2.32	1.73	3.12	2.50	1.86	3.35	3.03	1.48	6.21
Binge Drinking x MDE	**1.78**	**1.09**	**2.91**	0.85	0.50	1.44	1.23	0.44	3.41
Daily Smoking/Vaping	1.35	0.97	1.88	1.02	0.70	1.50	1.28	0.43	3.84
MDE	3.61	2.89	4.50	2.75	2.12	3.56	3.25	1.62	6.52
Daily Smoking/Vaping x MDE	**0.45**	**0.24**	**0.84**	**0.59**	**0.36**	**0.99**	0.89	0.22	3.62
Cannabis Use	1.36	0.96	1.91	1.12	0.73	1.73	3.04	1.13	8.20
MDE	2.94	2.34	3.71	2.51	1.96	3.21	4.60	2.49	8.49
Cannabis Use x MDE	0.90	0.49	1.64	0.73	0.36	1.46	**0.21**	**0.06**	**0.79**
Prescription Drug Abuse	2.72	1.66	4.45	2.65	1.54	4.57	6.37	2.43	16.68
MDE	3.06	2.47	3.81	2.56	2.02	3.25	4.29	2.47	7.45
Prescription Drug Abuse x MDE	**0.38**	**0.15**	**0.96**	**0.31**	**0.13**	**0.75**	**0.10**	**0.02**	**0.49**
Alcohol Use Disorder	1.63	1.01	2.62	1.33	0.75	2.38	0.84	0.24	2.91
MDE	2.69	2.12	3.41	2.42	1.88	3.11	3.15	1.69	5.87
Alcohol Use Disorder x MDE	1.13	0.52	2.48	0.79	0.33	1.87	1.26	0.28	5.74

Note: The table shows adjusted odds ratios (AORs) with 95% confidence intervals (CIs). Models adjust for socio-demographic characteristics, military status, and lifetime suicidality as described in Data Analysis. Significant interactions are shown in bold. MDE=major depressive episode.

Supplementary Table 6: Models of wave 1 substance use x "time since active duty" interactions on suicidal behaviors at wave 2

	Suicidal ideation			Suicide Plan			Suicide attempt		
	AOR	CI lower	CI upper	AOR	CI lower	CI upper	AOR	CI lower	CI upper
Binge Drinking	1.41	1.16	1.72	1.23	0.99	1.54	0.97	0.55	1.72
1-12 months	0.82	0.58	1.15	0.91	0.61	1.36	0.59	0.24	1.48
Binge Drinking x 1-12 months	1.00	0.61	1.64	0.98	0.55	1.76	1.89	0.41	8.72
Daily Smoking/Vaping	1.04	0.75	1.44	0.82	0.62	1.09	1.36	0.72	2.57
1-12 months	0.77	0.58	1.03	0.88	0.62	1.23	0.82	0.33	2.03
Daily Smoking/Vaping x 1-12 months	1.61	0.82	3.17	1.25	0.56	2.78	0.87	0.17	4.38
Cannabis Use	1.44	1.07	1.95	1.01	0.68	1.52	1.11	0.54	2.28
1-12 months	0.84	0.64	1.10	0.89	0.64	1.25	0.65	0.31	1.36
Cannabis Use x 1-12 months	1.00	0.46	2.19	1.22	0.50	2.98	2.47	0.30	20.04
Prescription Drug Abuse	2.36	1.38	4.01	1.73	1.00	2.98	1.27	0.52	3.07
1-12 months	0.85	0.65	1.11	0.95	0.69	1.30	0.60	0.29	1.24
Prescription Drug Abuse x 1-12 months	0.47	0.13	1.74	0.41	0.09	1.81	5.71	0.69	47.27
Alcohol Use Disorder	2.42	1.83	3.22	1.49	1.13	1.95	1.14	0.55	2.35
1-12 months	0.87	0.66	1.14	0.94	0.68	1.31	0.62	0.29	1.33
Alcohol Use Disorder x 1-12 months	0.62	0.31	1.27	0.74	0.32	1.75	2.69	0.42	17.20

Note: The table shows adjusted odds ratios (AORs) with 95% confidence intervals (CIs). Models adjust for socio-demographic characteristics, military status, and lifetime suicidality as described in Data Analysis. Significant interactions are shown in bold.

Supplementary Table 7: Interaction models of suicide attempt in the subgroup with past-12-month suicidal ideation at LS1 (n=1527)

Moderation by sex

	AOR	CI lower	CI upper	Chi-square	p value
Binge Drinking	1.03	0.50	2.15	0.01	0.93
Female	1.29	0.53	3.18	0.31	0.58
Binge Drinking x Female	1.26	0.33	4.83	0.11	0.74
Daily Smoking/Vaping	1.30	0.58	2.89	0.40	0.53
Female	1.44	0.64	3.21	0.78	0.38
Daily Smoking/Vaping x Female	0.83	0.17	4.20	0.05	0.83
Cannabis Use	0.59	0.23	1.50	1.23	0.27
Female	1.28	0.56	2.93	0.35	0.56
Cannabis Use x Female	1.49	0.24	9.09	0.18	0.67
Prescription Drug Abuse	0.93	0.33	2.64	0.02	0.89
Female	1.43	0.67	3.06	0.86	0.36
Prescription Drug Abuse x Female	0.79	0.08	7.78	0.04	0.84
Alcohol Use Disorder	0.99	0.40	2.44	0.00	0.98
Female	1.28	0.56	2.97	0.34	0.56
Alcohol Use Disorder x Female	1.41	0.26	7.72	0.16	0.69
**Moderation by depression**					
Binge Drinking	1.03	0.27	3.92	0.00	0.97
MDE	3.11	1.29	7.52	6.36	0.01
Binge Drinking x MDE	1.02	0.20	5.21	0.00	0.98
Daily Smoking/Vaping	0.48	0.11	2.05	0.99	0.32
MDE	2.60	1.16	5.80	5.40	0.02
Daily Smoking/Vaping x MDE	2.50	0.45	13.95	1.09	0.30

	AOR	CI lower	CI upper	Chi-square	p value
Cannabis Use	0.61	0.15	2.40	0.51	0.48
MDE	3.28	1.56	6.89	9.83	0.00
Cannabis Use x MDE	1.02	0.20	5.27	0.00	0.98
Prescription Drug Abuse	2.26	0.45	11.32	0.98	0.32
MDE	3.65	1.75	7.58	12.00	0.00
Prescription Drug Abuse x MDE	0.27	0.03	2.16	1.54	0.22
Alcohol Use Disorder	0.53	0.10	2.68	0.60	0.44
MDE	2.84	1.36	5.92	7.79	0.01
Alcohol Use Disorder x MDE	2.03	0.32	12.78	0.57	0.45
**Moderation by time since active duty**					
Binge Drinking	1.01	0.51	1.98	0.00	0.99
1-12 months	0.56	0.19	1.68	1.06	0.30
Binge Drinking x 1-12 months	2.04	0.35	11.91	0.62	0.43
Daily Smoking/Vaping	1.27	0.61	2.66	0.41	0.52
1-12 months	0.88	0.30	2.59	0.05	0.82
Daily Smoking/Vaping x 1-12 month	0.54	0.06	4.54	0.33	0.57
Cannabis Use	0.54	0.24	1.22	2.18	0.14
1-12 months	0.54	0.21	1.34	1.79	0.18
Cannabis Use x 1-12 months	5.22	0.56	48.40	2.12	0.15
Prescription Drug Abuse	0.52	0.18	1.50	1.46	0.23
1-12 months	0.53	0.21	1.35	1.77	0.18
Prescription Drug Abuse x 1-12 mon	9.08	0.83	99.06	3.27	0.07
Alcohol Use Disorder	0.95	0.43	2.12	0.02	0.90
1-12 months	0.60	0.22	1.62	1.02	0.31
Alcohol Use Disorder x 1-12 months	2.52	0.34	18.55	0.82	0.36

Note: The table shows adjusted odds ratios (AORs) with 95% confidence intervals (CIs). Models adjust for socio-demographic characteristics, military status, and lifetime suicidality as described in Data Analysis. Significant interactions are shown in bold. MDE=Major depressive edpisode.
